# Supplementary figures and images for: Developing lifestyle intervention program for pre-hypertensive patients; consensus building using a modified Delphi approach
Source: PLoS One. 2024 Oct 10;19(10):e0311766. doi: 10.1371/journal.pone.0311766 (PMC11469599; doi:10.1371/journal.pone.0311766)

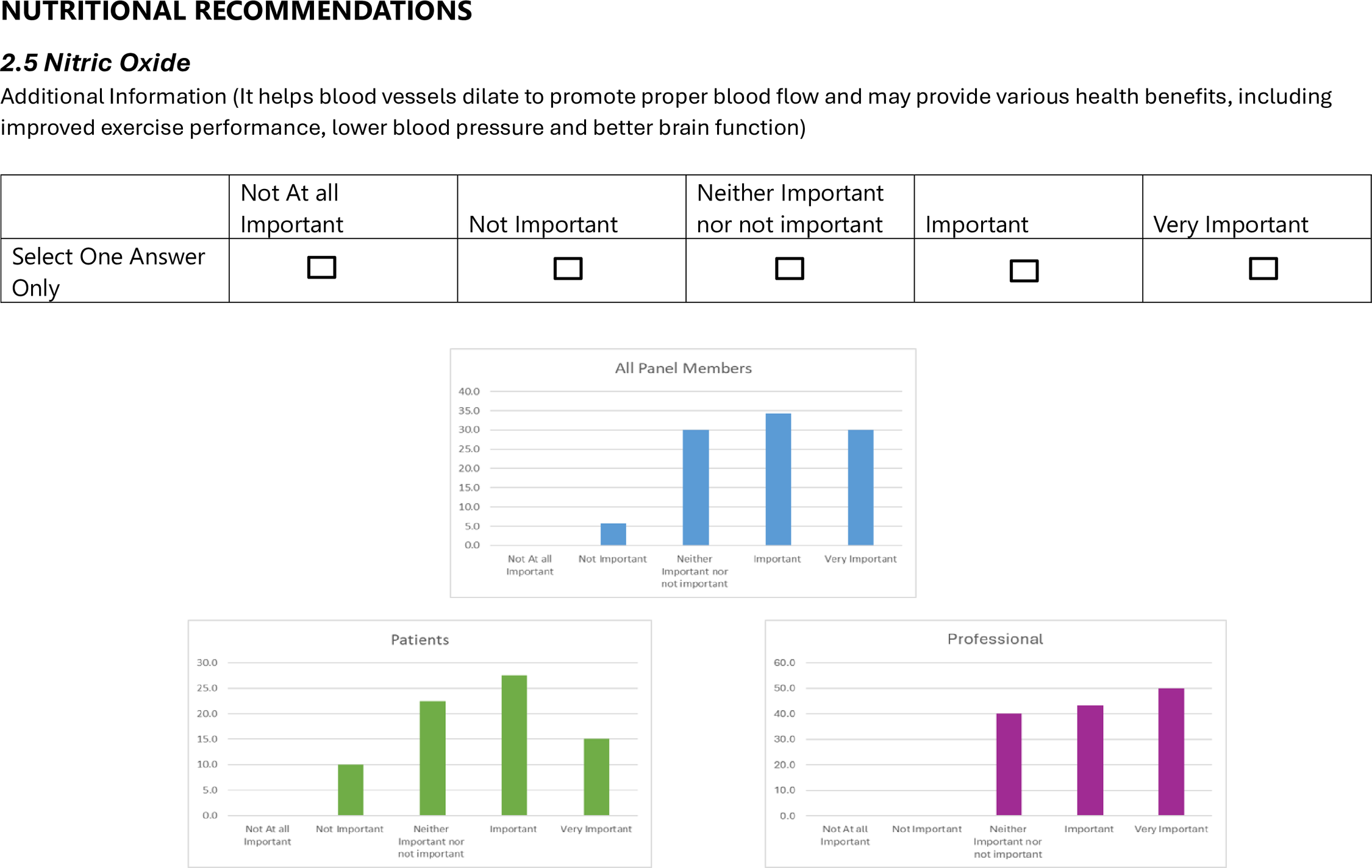

Supplement: S1 Fig — (TIF) [file pone.0311766.s002.tif]
