# Supplementary material for: Developing lifestyle intervention program for pre-hypertensive patients; consensus building using a modified Delphi approach
Source: PLoS One. 2024 Oct 10;19(10):e0311766. doi: 10.1371/journal.pone.0311766 (PMC11469599; doi:10.1371/journal.pone.0311766)
Supplement: S2 Table — (DOCX) [file pone.0311766.s004.docx]

Supplementary Table 2: Consensus percentage for Patients and Professional for Dietary Recommendation Items

| **Total Dietary Recommendations items** | **Round 1 (n=70)** | | | **Round 2 (n=63)** | | | **Round 3 (n=51)** | | |
| --- | --- | --- | --- | --- | --- | --- | --- | --- | --- |
|  | Patients | Professional | Overall | Patients | Professional | Overall | Patients | Professional | Overall |
| 1. Olive Oil | 66.7 | 82.5 | 75.7 | 71.4 | 88.6 | 81.0 | 76.0 | 80.8 | 78.4 |
| 1. Green Tea | 76.7 | 75.0 | 75.7 | 78.6 | 80.0 | 79.4 | 76.0 | 76.9 | 76.5 |
| 1. Omega-3 fat alpha-linolenic acid (ALA) | 80.0 | 90.0 | 85.7 | 82.1 | 88.6 | 85.7 | 80.0 | 84.6 | 82.4 |
| 1. High Intake of Dietary Potassium | 50.0 | 70.0 | 61.4 | 50.0 | 74.3 | 63.5 | 44.0 | 65.4 | 54.9 |
| 1. Nitric Oxide | 56.7 | 70.0 | 64.3 | 60.7 | 77.1 | 69.8 | 60.0 | 73.1 | 66.7 |
| 1. Fruits Consumption | 66.7 | 75.0 | 71.4 | 78.6 | 88.6 | 84.1 | 68.0 | 88.5 | 78.4 |
| 1. Vegetables Consumptions | 70.0 | 82.5 | 77.1 | 71.4 | 94.3 | 84.1 | 72.0 | 88.5 | 80.4 |
| 1. Nuts Consumptions | 70.0 | 75.0 | 72.9 | 67.9 | 85.7 | 77.8 | 68.0 | 80.8 | 74.5 |
| 1. Low Fat Dairy Products | N/A | | | 75.0 | 88.6 | 82.5 | 80.0 | 88.5 | 84.3 |
| 1. Whole Grains | N/A | | | 64.3 | 80.0 | 73.0 | 64.0 | 76.9 | 70.6 |
| 1. Oats | N/A | | | 57.1 | 80.0 | 69.8 | 56.0 | 76.9 | 66.7 |
| 1. Barley | N/A | | | 60.7 | 80.0 | 71.4 | 60.0 | 84.6 | 72.5 |
| 1. Cereals | N/A | | | 92.9 | 82.9 | 87.3 | 92.0 | 80.8 | 86.3 |
| 1. Pre-Breakfast | N/A | | | 17.9 | 51.4 | 36.5 | 20.0 | 57.7 | 39.2 |
