# Supplementary material for: Developing lifestyle intervention program for pre-hypertensive patients; consensus building using a modified Delphi approach
Source: PLoS One. 2024 Oct 10;19(10):e0311766. doi: 10.1371/journal.pone.0311766 (PMC11469599; doi:10.1371/journal.pone.0311766)
Supplement: S3 Table — (DOCX) [file pone.0311766.s005.docx]

Supplementary Table 3: Consensus percentage for Patients and Professional for Exercise Recommendation Items

| **Total Exercise Recommendations items** | **Round 1 (n=70)** | | | **Round 2 (n=63)** | | | **Round 3 (n=51)** | | |
| --- | --- | --- | --- | --- | --- | --- | --- | --- | --- |
|  | Patients | Professional | Overall | Patients | Professional | Overall | Patients | Professional | Overall |
| 1. Yoga Therapy | 73.3 | 67.5 | 70.0 | 89.3 | 77.1 | 82.5 | 92.0 | 73.1 | 82.4 |
| 1. Isometric Hand Grip Exercises | 63.3 | 62.5 | 62.9 | 67.9 | 74.3 | 71.4 | 72.0 | 80.8 | 76.5 |
| 1. Aerobic Exercise | 73.3 | 77.5 | 75.7 | 71.4 | 77.1 | 74.6 | 68.0 | 88.5 | 78.4 |
| 1. Stretching Exercises | 63.3 | 70.0 | 67.1 | 60.7 | 77.1 | 69.8 | 60.0 | 80.8 | 70.6 |
| 1. Resistance Exercises | 80.0 | 77.5 | 78.6 | 78.6 | 80.0 | 79.4 | 80.0 | 76.9 | 78.4 |
| 1. Walking | 70.0 | 75.0 | 72.9 | 67.9 | 74.3 | 71.4 | 68.0 | 76.9 | 72.5 |
| 1. Commuting | N/A | | | 85.7 | 48.6 | 65.1 | 84.0 | 46.2 | 64.7 |
| 1. Brisk Walking | N/A | | | 71.4 | 82.9 | 77.8 | 76.0 | 84.6 | 80.4 |
| 1. Desk treadmilling | N/A | | | 64.3 | 74.3 | 69.8 | 68.0 | 84.6 | 76.5 |
| 1. Swimming | N/A | | | 57.1 | 34.3 | 44.4 | 52.0 | 34.6 | 43.1 |
| 1. Hiking | N/A | | | 53.6 | 65.7 | 60.3 | 52.0 | 73.1 | 62.7 |
| 1. High Intensity Interval Training | N/A | | | 60.7 | 80.0 | 71.4 | 68.0 | 96.2 | 82.4 |
| 1. Circuit Training | N/A | | | 89.3 | 57.1 | 71.4 | 88.0 | 80.8 | 84.3 |
