# Supplementary material for: Developing lifestyle intervention program for pre-hypertensive patients; consensus building using a modified Delphi approach
Source: PLoS One. 2024 Oct 10;19(10):e0311766. doi: 10.1371/journal.pone.0311766 (PMC11469599; doi:10.1371/journal.pone.0311766)
Supplement: Attachment — Submitted filename: Response to reviewers.docx [file pone.0311766.s007.docx]

**PONE-D-24-23230**

**Developing lifestyle intervention program for pre-hypertensive patients; consensus building using a modified Delphi approach**

| **Comments from reviewer** | **Response from author** |
| --- | --- |
| Please ensure that your manuscript meets PLOS ONE's style requirements, including those for file naming. | The manuscript has been formatted to meet PLOS ONE's style requirements, including those for file naming. |
| We note that the grant information you provided in the ‘Funding Information’ and ‘Financial Disclosure’ sections do not match. | The grant information provided in the ‘Funding Information’ and ‘Financial Disclosure’ sections now match. |
| Your ethics statement should only appear in the Methods section of your manuscript. If your ethics statement is written in any section besides the Methods, please move it to the Methods section and delete it from any other section. Please ensure that your ethics statement is included in your manuscript, as the ethics statement entered into the online submission form will not be published alongside your manuscript. | Ethics statement is not added in the methodology section and removed from other parts of the manuscript.  Page: 5  Line no. 100 |
| Please include captions for your Supporting Information files at the end of your manuscript, and update any in-text citations to match accordingly. | The captions for the Supporting Information files have been included at the end of the manuscript, and all in-text citations have been updated accordingly.  Page: 28 |
| Modify the abstract - shorten the background, and in results/conclusion section include what novel implications the study adds. You may want to include the what educational, dietary, and exercise components came out of the study that were different from the earlier standard of care. | Abstract has been updated as per recommendations. Background is shorter now and results and conclusion sections are also updated.  Page: 2, 3  Line no. 15 – 19, 38 – 50. |
| In the discussion section, please clearly mention what the final model includes, what is different from the earlier known standard - you might include some references for the previously know and used Standard of Care. | The discussion section has been modified, and additional text has been added to clarify the novelty of the final model compared to previous literature. References to the previously known and used Standard of Care have also been included.  Page: 16 – 19 |
