# Supplementary material for: Developing lifestyle intervention program for pre-hypertensive patients; consensus building using a modified Delphi approach
Source: PLoS One. 2024 Oct 10;19(10):e0311766. doi: 10.1371/journal.pone.0311766 (PMC11469599; doi:10.1371/journal.pone.0311766)
Supplement: Attachment — Submitted filename: Response to reviewers.docx [file pone.0311766.s008.docx]

**PONE-D-24-23230**

**Developing lifestyle intervention program for pre-hypertensive patients; consensus building using a modified Delphi approach**

| **Comments from reviewer** | **Response from author** |
| --- | --- |
| Journal Requirements:  Please review your reference list to ensure that it is complete and correct. If you have cited papers that have been retracted, please include the rationale for doing so in the manuscript text, or remove these references and replace them with relevant current references. Any changes to the reference list should be mentioned in the rebuttal letter that accompanies your revised manuscript. If you need to cite a retracted article, indicate the article’s retracted status in the References list and also include a citation and full reference for the retraction notice. | The reference list is complete and is not being updated in the revised manuscript.  No retracted paper has been cited in this manuscript to date. |
| Thank you for submitting your valuable work i would like to recommend adding some details on secondary hypertension on your introduction or discussion for example patients with renal hypertension and weather it had been addressed before in other studies or not and if the same recommendations is applicable to patients with essential hypertension. | Thank you for your valuable feedback and thoughtful recommendation. We appreciate your suggestion to include information on secondary hypertension, particularly in relation to conditions such as renal hypertension, and whether similar recommendations have been addressed in previous studies.  As the primary focus of our study is prehypertension, we would like to clarify that the lifestyle modifications recommended through our Delphi study are specifically designed for pre-hypertensive patients. According to JNC 7 guidelines for managing high blood pressure, prehypertensive patients are ideal candidates for lifestyle modification, potentially bypassing the need for pharmacological intervention. In contrast, the pathophysiology of essential and secondary hypertension is more complex and often associated with multiple comorbid factors. For example, renal hypertension may require specific medical interventions to address underlying kidney issues, whereas essential hypertension often necessitates pharmacological treatment.  We hope this explanation addresses your concern. |
